# Development of a Human Cranial Bone Surrogate for Impact Studies

**DOI:** 10.3389/fbioe.2013.00013

**Published:** 2013-10-24

**Authors:** Jack C. Roberts, Andrew C. Merkle, Catherine M. Carneal, Liming M. Voo, Matthew S. Johannes, Jeff M. Paulson, Sara Tankard, O. Manny Uy

**Affiliations:** ^1^Applied Physics Laboratory, The Johns Hopkins University, Laurel, MD, USA; ^2^The Johns Hopkins University, Baltimore, MD, USA

**Keywords:** cranial bone, surrogate, simulant, testing, material, impact

## Abstract

In order to replicate the fracture behavior of the intact human skull under impact it becomes necessary to develop a material having the mechanical properties of cranial bone. The most important properties to replicate in a surrogate human skull were found to be the fracture toughness and tensile strength of the cranial tables as well as the bending strength of the three-layer (inner table-diplöe-outer table) architecture of the human skull. The materials selected to represent the surrogate cranial tables consisted of two different epoxy resins systems with random milled glass fiber to enhance the strength and stiffness and the materials to represent the surrogate diplöe consisted of three low density foams. Forty-one three-point bending fracture toughness tests were performed on nine material combinations. The materials that best represented the fracture toughness of cranial tables were then selected and formed into tensile samples and tested. These materials were then used with the two surrogate diplöe foam materials to create the three-layer surrogate cranial bone samples for three-point bending tests. Drop tower tests were performed on flat samples created from these materials and the fracture patterns were very similar to the linear fractures in pendulum impacts of intact human skulls, previously reported in the literature. The surrogate cranial tables had the quasi-static fracture toughness and tensile strength of 2.5 MPa√ m and 53 ± 4.9 MPa, respectively, while the same properties of human compact bone were 3.1 ± 1.8 MPa√ m and 68 ± 18 MPa, respectively. The cranial surrogate had a quasi-static bending strength of 68 ± 5.7 MPa, while that of cranial bone was 82 ± 26 MPa. This material/design is currently being used to construct spherical shell samples for drop tower and ballistic tests.

## Introduction

A number of studies have been performed using post-mortem human surrogate (PMHS) specimen to study the effect of blunt impact on pressures and displacements in the brain (Hardy et al., 1997, 2001), determine skull failure thresholds and characterize skull fracture patterns (Gurdjian et al., 1949; Melvin et al., 1969; Hodgson et al., 1970; Schneider and Nahum, 1972; Sarron et al., 2004; Hart, 2005; Delye et al., 2007; Verschueren et al., 2007; Raymond et al., 2009). Due to the complexity of these PMHS experiments along with the inherent likelihood of significant specimen variability, it would be prudent to have a skull surrogate which can represent the stress, vibration, and fracture characteristics of human cranial bone in a repeatable manner. This surrogate could then be used to assess the likelihood of skull fracture during impact and ultimately influence the design of mitigation techniques. In order to develop this skull surrogate material, it becomes necessary to consider the architecture of a human skull. The skull consists of an energy absorbing porous layer (diplöe) sandwiched between higher strength and stiffness denser layers (hereafter referred to as cranial tables). Gurdjian et al. (1950) showed that in human skulls with stresscoat applied in the area surrounding the impact point, if impact is not severe enough to cause fracture at the boundary of the concave area of the skull (tensile stress on the inside of the skull is not exceeded), the skull will rebound. However, areas away from the impact point that have experienced convex curvature can exceed the tensile strength on the outside of the skull and fracture. This indicates that the bending stiffness and bending strength of the skull and tensile strength of the outer fiber of the skull, i.e., cranial tables, are important in skull fracture. Besides linear fractures that occur toward or away from the impact point, there are concentric fractures, that result in a sinking or Cupule fracture in the immediate impact area (Gurdjian et al., 1950; Sarron et al., 2000; Hart, 2005; Raymond et al., 2009). In recent studies by Thali (2003) and Thai (2003) a skin-skull-brain model was developed that produced fracture patterns in the skulls similar to those seen in humans with gunshot wounds to the head. Although a three-layer (Tabula interna-diplöe-Tabula externa) polyurethane material was used in these studies, no effort was made to develop a skull model to have the mechanical properties of human cranial bone. In any impact scenario failure of cranial bone may be preceded by fracture of the cranial tables, followed by bending of cranial bone until it fails. Therefore, the fracture toughness and tensile strength of the cranial tables along with bending stiffness and bending strength of cranial bone may be some of the governing factors leading to cranial bone failure.

The purpose of this study was to develop cranial surrogate bone with the bending strength of human cranial bone and with cranial tables having the fracture toughness and tensile strength of human compact bone. Candidate surrogate cranial table materials were selected and fracture toughness and tensile tests performed to see how well they approximate the tensile and fracture properties of human cranial bone tables in the literature. Surrogate foam materials representing the diplöe were selected and full three-layer cranial surrogates were constructed using the cranial surrogate table materials that best represent human compact bone. Three-point bending tests were performed on the surrogate materials and the best materials were selected for drop tower testing. Flat panel samples were then molded from these materials and drop tower tests were performed.

## Materials and Methods

**Figure 1:**
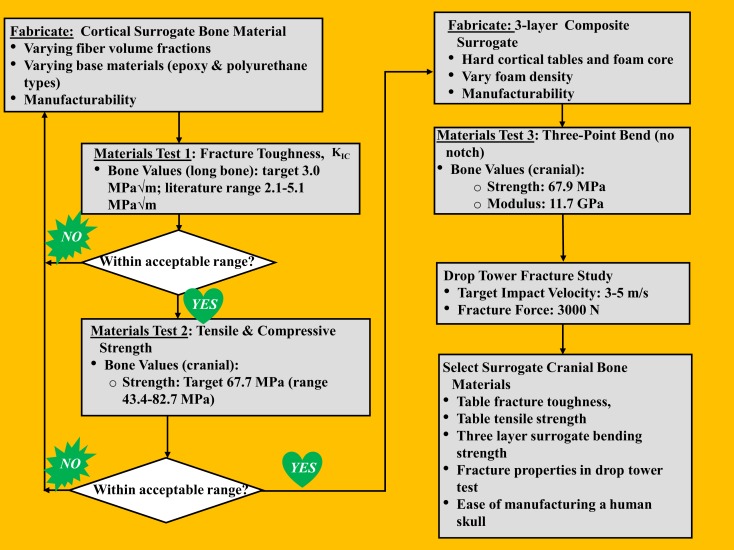
**Human skull surrogate development process**.

A stepwise process was followed to develop a skull surrogate complete with key material properties that govern fracture characteristics of the skull, shown in the flowchart in Figure 1, as will be explained in this section. For the cranial tables, targeted properties of interest were the fracture toughness (*K*_IC_) and tensile strength/modulus, which are indicative of a material’s resistance to crack propagation and the maximum bending radius a material can sustain before failing in tension, respectively. Cranial table compositions that most closely targeted real skull values for fracture toughness and tensile strength were used to sandwich a variety of diplöe-simulating foam materials, thus creating a three-layer composite structure. These sandwich-structured skull surrogates were subjected to a second round of testing, where the bending strength, force to fracture, and impact fracture patterns were used for the final materials selection for incorporation into the flat-plate test rig.

Literature values for the bending modulus, bending strength, tensile strength, and the mode-I fracture toughness (*K*_IC_) and target values of both human cranial and cortical bone are shown in Table 1. Although the tensile strength of human cranial tables can be found in the literature, apparently the fracture toughness of human cranial bone has not been measured. Since the cranial tables just consist of cortical bone, the fracture toughness of cortical bone in the femur was chosen to replicate in the surrogate cranial bone table values. The values for average thickness of cranial tables and cranial bone from the literature were found to be 1.54 and 6.5 mm, respectively (Hubbard, 1971). The bending modulus and bending strength of cranial bone in Hubbard (1971) had to be calculated from the sectional stiffness and maximum moment divided by beam width based on the raw data of individual samples.

**Table 1:** **Mechanical properties of human cranial and cortical bone along with achieved surrogate values**.

Bone properties	Fracture toughness *K*_IC_ MPa√ m	Tensile strength (MPa)	Bending modulus (GPa)	Bending strength (MPa)
Human cranial bone (Hubbard, 1971; Wood, 1971)	–	–	11.73 ± 0.95	82.0 ± 25.5
Human cranial table (Robbins and Wood, 1969; McElhaney et al., 1970; Wood, 1971)	–	67.73 ± 17.8	–	–
Human cortical bone (longitudinal) (Norman et al., 1996; Vashishth et al., 1997; Wang and Puram, 2004)	3.07 ± 1.75	–	–	–
Surrogate table (30% milled GL fiber)	2.5	53 ± 4.88		
Cranial surrogate	–	–	–	67.9 ± 5.67

All surrogate cranial table samples were fabricated using either the resins EPON 815C/EPIKURE 3234 or EPON 862/EPIKURE 3274 (Miller-Stephenson Chemical Co.) The test samples included neat epoxy or epoxy with randomly oriented milled 1.6 mm glass fibers (Fibre Glast Developments Corporation). A zirconium coupling agent (Kenrich Petrochemicals, Inc. KZ-55, 0.5% by weight) was added to the mix to suspend and disburse the milled glass fibers as uniformly as possible and an anti-foam surfactant (Huntsman Vantico RP-806, 0.2% by weight) was added to expedite degassing and improve resin flow and wetting. The final mixtures were degassed for 6 min and then poured into rubber molds that were fabricated to conform to the beam samples in the ASTM testing standards. The samples were cured under pressure (414 kPa) for 12 h before de-molding, followed by an ambient temperature for a minimum of 24 h prior to testing.

The surrogate diplöe materials selected were three urethane foams of differing density. The foams consisted of U.S. foam 16# with an as formed density of 0.29 g/cc (U.S. Composites), a modified (vacuum degassing to create higher as formed density – 1.03 g/cc) U.S. foam 16# (U.S. Composites) and TC-812 with a formed density of 0.64 g/cc (BJB Enterprises, Inc.). The densities of these materials were determined from the samples that were cured in the three-layer configuration. The compressive and shear strengths for US foam 16# were 4 and 1.59 MPa, respectively, and for TC-812 were 21 and 18 MPa, respectively. The process to fabricate the surrogate cranial bone samples starts with inserting the two glass/epoxy composite plates (inner and outer surrogate cranial tables) into an aluminum mold with the abraded side facing inward. The skull surrogate diplöe material, which is a two-part expanding urethane foam was injected between the two epoxy plates and allowed to expand and cure for a minimum of 60 min before de-molding. Two different foam materials were used for the diplöe to see the degree of difficulty they would present when creating flat samples and to asses any difference in the bending properties of the three-layer composites surrogate bone samples. One of the more important aspects of this study was to assure that when the resin and weight percent glass filler had been established, the three-layered composite surrogate bone material could be manufactured in a complex anatomically correct human skull shape. Too much entrapped air in differing thickness sections of the surrogate skull would result in weakened sections that would prematurely fail, hence the importance of term “manufacturability” in Figure 1.

An impact method was desired to evaluate risk of skull fracture using a conceptual helmet system in a configuration that is readily employable and does not rely on local curvature. Therefore, the method employed was to design a test fixture that would use a flat skull surrogate sample in a drop tower test. As a first test configuration this design would allow for ease of manufacture and more repeatable results when only comparing one sample material to another.

### Testing

#### Fracture toughness and tensile

ASTM D 5045, *Standard Test Methods for Plane-Strain Fracture Toughness and Strain Energy Release Rate of Plastic Materials*^1^ was used for the glass/epoxy surrogate cranial table materials. In order to have a valid measurement for fracture toughness, the following equalities must be met,

(1) $$B,\;a,\;\left(W-a\right)>2.5{\left(\frac{K_{IC}}{\sigma_{y}}\right)}^{2}$$

and,

(2) $$0.45<\frac{a}{W}<0.55$$

In the above equation, *B* is the sample thickness, *W* is the sample height, and *A* is the notch depth, while σ*_y_* is the maximum stress at failure and *K*_IC_ is the mode-I critical stress intensity factor. The target values for the stress intensity factor (fracture toughness) and tensile strength were 3.07 MPa√ m and 67.73 MPa, respectively, see Table 1. Using the above values and designing a specimen that would be practical in terms of size, the final dimensions for the sample were, *B* = 15 mm, *a* = 15 mm, and *W* = 30 mm.

**Figure 2:**
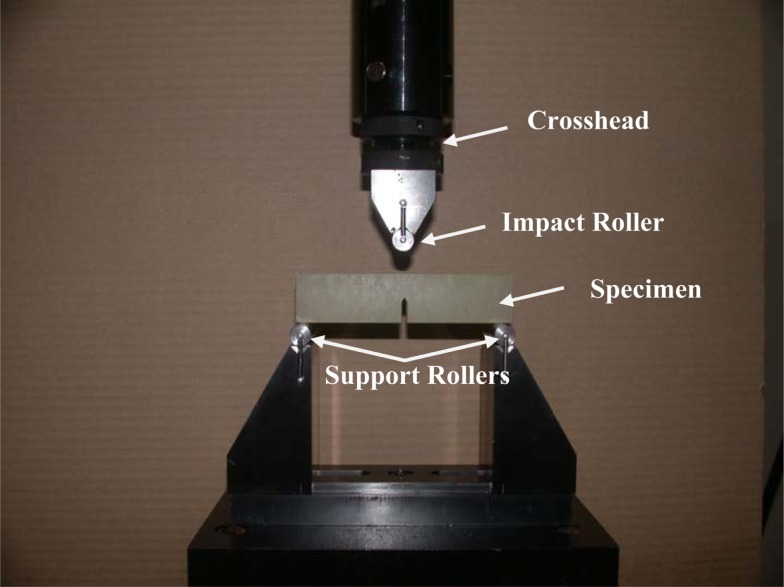
**Three-point bending test set-up to determine fracture toughness according to ASTM D 5405**. The cross-head was run at a constant rate and the loading data was sampled from an in-line load cell.

An Instron 8821S hydraulic servo controlled machine was used for testing. A three-point bend configuration was used with the specimen placed on the support fixture (Wyoming Fixture Model CU-FL) which was mounted on a load cell (Dynacell, Model # 2527-101), see Figure 2. The loading rate was nominally set at 10 mm/s over a drive amplitude of 30 mm and data was collected at 5 kHz. The fracture test was performed and the loading/deflection data was collected. The equations used to get *K*_IC_ (Srawley, 1976) are shown as,

(3) $$K_{IC}=\left(\frac{P}{BW^{1.2}}\right)f\left(x\right)$$

And for (0 < *x* < 1):

(4) $$f{\left(x\right)}_{c}=6x^{\frac{1}{2}}\frac{\left[1.99-x\left(1-x\right)\left(2.15-3.93x+2.7x^{2}\right)\right]}{\left(1+2x\right){\left(1-x\right)}^{\frac{3}{2}}}$$

where *P* is the load at failure, *A*, *B*, and *W* are as previously defined and *x* = *A*/*W*.

Surrogate cranial table tensile strength and modulus were determined by ASTM D 638, *Standard Test Method for Tensile Properties of Plastics*^2^. The preferred sample thickness for tensile testing was 3.2 ± 0.4 mm and the overall length of the dog bone samples was 165 mm. They were pulled in tension at a rate of 2.54 mm/s and failed as a linear elastic fashion.

#### Bending

**Figure 3:**
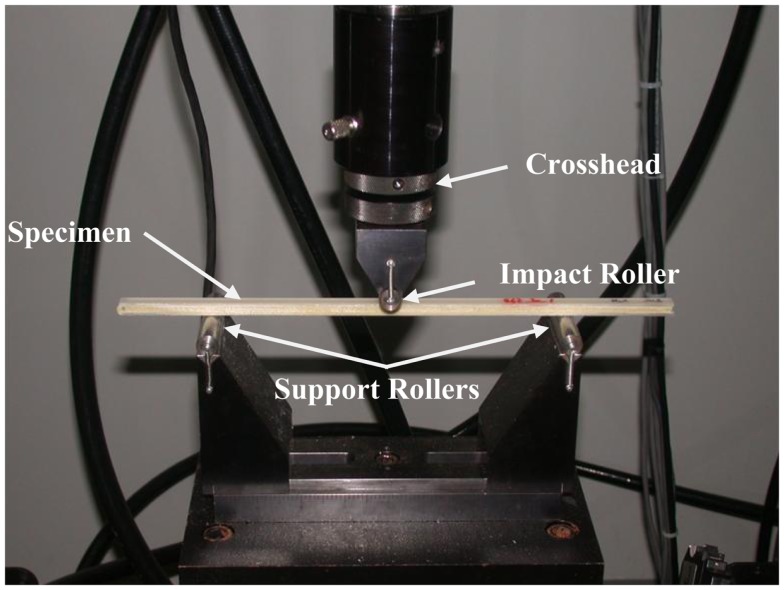
**Three-point bending test set-up to determine flexural strength of the three-layer surrogate cranial bone samples according to ASTM D 790**. The cross-head was run at a constant rate and the loading data was sampled from an in-line load cell.

Based on male and female anatomical data, it was decided to standardize on a total skull surrogate thickness of 8 mm, with the inner and outer cranial tables either 2 or 2.2 mm and the resulting diplöe thickness of 4 or 3.6 mm, respectively. The determination of bending strength and modulus for the full three-layer composite was performed according to ASTM D 790, *Standard Test Methods for Flexural Properties of Unreinforced and Reinforced Plastics and Electrical Insulating Materials*^3^. Based on this standard and geometric restrictions imposed by the three-point bend test fixture, the resulting specimens were constructed to be 8 mm by 8 mm in cross section and have a length of not less than 250 mm. As in the fracture toughness test, the bending tests were performed at 10 mm/s. The Instron set-up was identical to that of the fracture toughness tests, but with samples rotated 90° to that in the fracture toughness test and no notch in the sample, see Figure 3. The equations used to calculate the stress at failure (strength), are as shown below (ASTM D 790),

(5) $$\sigma_{f}=\frac{3PL}{2b_{1}d_{1}^{2}}$$

where *P* is the load at failure, *L* = 250 mm, *b*_1_ = 8 mm, *d*_1_ = 8 mm, and *D* = deflection at the center of the beam. Then the tangent modulus of elasticity or bending stiffness can be given by,

(6) $$E_{b}=\frac{L^{3}m}{b_{1}4d_{1}^{3}}$$

where *E*_b_ is the modulus in bending, *m* is the slope of the tangent to the initial straight-line portion of the load-deflection curve and *L*, *b*_1_, and *d*_1_ are as given above.

#### Flat panel drop tower

**Figure 4:**
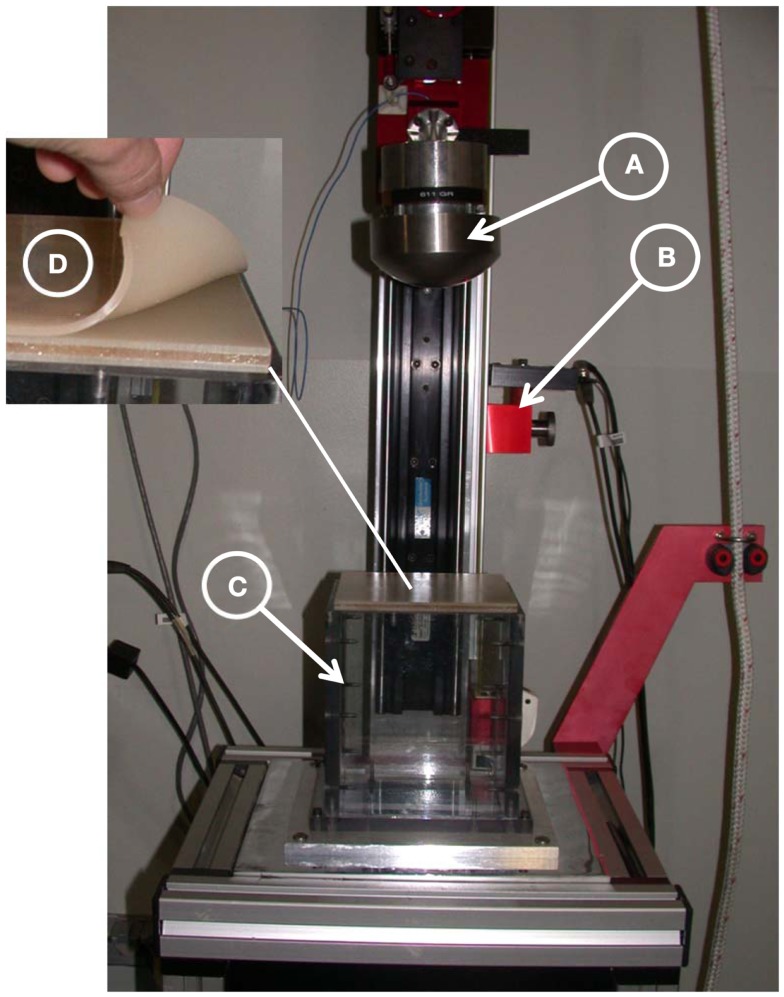
**Drop tower test set-up**. **(A)** A 10-cm spherical steel impactor instrumented with accelerometer, **(B)** fiber-optic gate, **(C)** hollow rectangular enclosure filled with Sylgard gel, and **(D)** three-layer cranial surrogate flat-plate sample with simulated skin.

A hollow rectangular enclosure, with one side open to allow attachment of the flat surrogate samples, was designed in polycarbonate. The enclosure was filled with silicone gel (Sylgard 527, Dow Corning), a material used by a number of investigators in brain trauma studies (Hodgson et al., 1970; Thibault et al., 1987; Meaney et al., 1995; Brands et al., 1999; Ivarsson et al., 2000, 2002; Bradshaw et al., 2001) and then the surrogate skull plates were placed on top and secured via compression around the edges, see Figure 4. Controlled impact conditions for the test (impactor mass and energy) were based on those used on fresh PMHS skull fracture studies by Hodgson et al. (1970). A layer of XP-656 silicone (Silicone, Inc.), 2.54 mm thick with a Shore hardness of 9 ± 3, was placed over the surrogate as a skin simulant. The fracture characteristics of the panels were then compared to skull fracture in the literature.

### Post-processing

One-way analysis of variance was performed (JMP 10 Pro software, SAS Institute, Inc., Cary, NC, USA) on the cranial materials to determine whether the tensile strength and modulus of the surrogate Table materials and the bending strength and modulus of the surrogate cranial bone supported the hypothesis that there were no differences between the cranial materials. For nearly 25 years, this statistical discovery software from SAS has been the tool of choice for scientists, engineers and other data explorers in almost every industry and government sector.

## Results and Discussion

### Fracture toughness and tensile testing of surrogate cranial table materials

**Figure 5:**
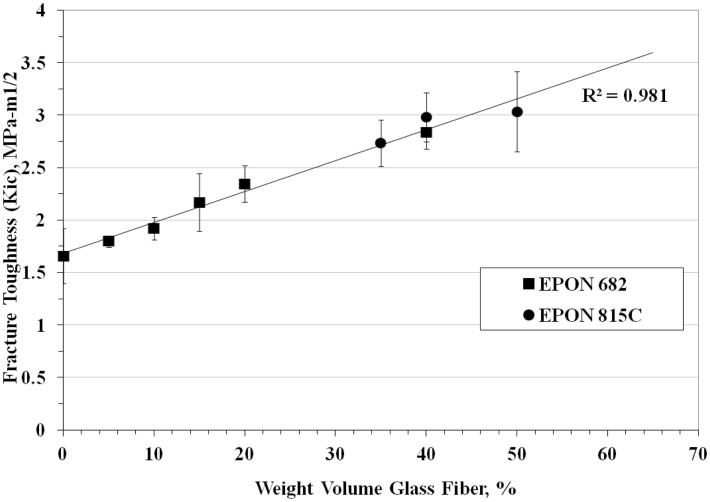
**Fracture toughness of surrogate cranial tables for different percentages of randomly oriented milled glass fiber**.

Fracture toughness tests were performed on 41 combinations of materials and Figure 5 is a plot of the fracture toughness versus weight percent fiber. The equation for fracture toughness as a function of weight percent milled glass fiber was found to be,

(7) $$K_{IC}=0.212W_{GL}+1.37\left(R^{2}=0.981\right)$$

where, *K*_IC_ is the fracture toughness and *W*_GL_ is the weight percent milled glass fiber. The values of *K*_IC_ varied from 1.66 ± 0.26 MPa√ m at zero weight percent milled glass fiber (resin only) to 3.03 ± 0.38 MPa√ m at 50 weight percent milled glass fiber. It should be noted that the type of resin used (EPON 815C or 862), had little effect on the average value of fracture toughness. This is important, because the viscosity of EPON 862 was higher than that of 815C, so as the percent glass filler increased toward 50 weight percent it became increasingly difficult to mix and degas the samples. If EPON 862 were used in molding the cranial tables of the actual cranial surrogates (with multiple curved surface), the inability to mold reproducible cranial table materials would result in surrogate skulls that would not adequately represent fracture properties of human skulls. Since the type of resin had little or no effect on fracture toughness and the fact that EPON 815C is more workable than EPON 862 with the higher weight percent milled glass fiber, EPON 815C was selected as the resin system to be used in all other tests. Therefore, based on the ability to adequately mix the materials to achieve a reproducible value of fracture toughness for the cranial table materials, especially for the varying thickness, curved surrogate skull geometry, the milled glass fiber content will be limited to 30–40 weight percent. This weight percent glass filler would result in a surrogate cranial table with a fracture toughness of 2.5–3.0 MPa√ m compared to the average human cranial table fracture toughness of 3.07 ± 1.75 MPa√ m. The average tensile strengths determined for 35 and 40% milled glass fiber form three test samples were 53.8 ± 4.11 and 52.43 ± 6.11 MPa, respectively, which puts them both in the range of values targeted for human cortical bone, i.e., 67.73 ± 17.8 MPa. In order to prevent “clumping” of the milled fiber in the fracture toughness and tensile group samples, it was necessary to introduce a zirconium coupling agent to suspend and disburse the milled glass fibers. In addition to this, the anti-foam surfactant was added to expedite degassing and improve resin flow and wetting. This worked well for both the fracture toughness and tensile samples.

### Three-point bending of surrogate cranial bone

Due to the fact that the higher percentage of fiber causes problems in sample molding, 30% glass fiber was used for the cranial table materials in the cranial surrogate three-point bending tests. As previously mentioned, two different foam materials were used for the simulated diplöe to see if the type of foam affected the bending strength of the cranial surrogate bone material. The flat test samples consisted of the foam material sandwiched between the inner and outer 2 mm thick cranial table material (EPON 815C + 30% milled glass fiber). The three-point bending strength and modulus for three samples of cranial table material with, US Foam 16# was 67.3 ± 4.5 MPa and 2.89 ± 0.22 GPa, and with BJB-TC 812 foam was 68.4 ± 7.7 MPa and 3.08 ± 0.15 GPa, respectively. These values are in the range of the bending strength of human cranial bone, which, from Table 1 is 82 ± 25.5 MPa. However, the average bending modulus for surrogate cranial bone from all six samples tested was 2.98 ± 0.20 GPa compared to 11.73 ± 0.95 MPa for human, therefore the resulting material would be somewhat more compliant than actual cranial bone. The analysis of variance of the tensile strength and modulus for the cranial table materials, as well as for the bending strength and modulus for the cranial bone materials, did not result in statistical significance where *p*-value > 0.05. The data did not therefore support the rejection of the null hypothesis, i.e., no difference in the cranial materials. Although the samples sizes were small, in most cases the *p*-values were so large (>0.5) that one could conclude that not many samples were required. The targeted values for the surrogate cranial tables and for overall bending of the three-layer structure are shown in Table 1 and compared to human cranial bone literature values.

### Flat panel drop tower testing

The drop tower tests were performed on three down-selected cranial surrogate material combinations based on the results of the fracture toughness, tensile and three-point bending tests. The materials selected were: (A) EPON 815C + 30% glass (2 mm thick) with a TC 812 foam core (4 mm thick), (B) EPON 815C + 30% glass (2 mm thick) with a US 16# foam core (4 mm thick) and (C) EPON 815C + 30% glass (2 mm thick) with a modified US 16# foam core (4 mm thick).

The average force to fracture for three samples materials of each of the materials tested (A, B, and C), were: 1956 ± 153, 1616 ± 218, and 1712 ± 338 N, respectively. Three samples of each material were tested. Although material A had the highest average force to fracture, each set of samples produced enough variation that the performance was deemed comparable. The variance may be due to a number of things, among them, the processing and molding of the samples, the adherence of the surrogate cranial tables to the diplöe and the type of fractures that are initiated in the surrogate cranial tables. The average force to fracture of all materials in the flat plates in the drop tower test was 1650 N, which considerably lower than the average force to fracture an intact human skull in both the protected and unprotected configuration under impact conditions that vary from drop tower to ballistic impact (Nahum et al., 1968; Melvin et al., 1969; Hodgson et al., 1970; Hubbard, 1971; Schneider and Nahum, 1972; Yoganandan et al., 1995; Sarron et al., 2004; Hart, 2005; Delye et al., 2007; Verschueren et al., 2007; Raymond et al., 2009). This number was 7055 ± 4070 N and depended on the nature of tissue preparation (embalmed or fresh), the geometry of the impactor, the rates of impact and what the region of the skull was being impacted. Because there is such a large variation in force to fracture the intact human skull, when designing a constant thickness skull model, the best practice would be to use a design fracture load to cover most areas of the skull under most conditions. Therefore, the best design fracture load may be a number corresponding to that of 1-SD below the mean, or about 3000 N. It should also be noted that this was a drop tower test on a flat plate, *not* a multi-radii of curvature skull. The addition curvature would likely increase the average force to fracture. Initial estimates predict that the effects of curvature would increase the fracture load by a factor of 2 to 3, which would put the fracture loads seen in the flat-plate surrogate samples into the same range as one standard deviation below the mean fracture load seen for intact human skulls.

**Figure 6:**
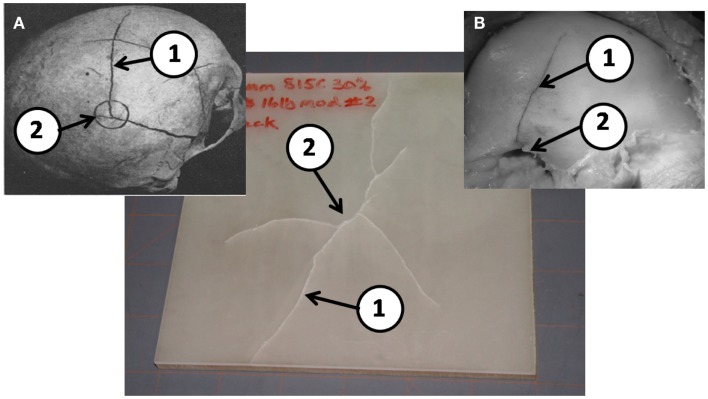
**A typical post drop tower test sample of EPON 815C with 30% milled glass fiber cranial tables and a 16# modified foam surrogate diplöe**. **(A)** Results from Gurdjian et al. (1950) and **(B)** from Delye et al. (2007) (reproduced with permission of copyright owners). Note the ductile nature of the linear fractures (1) away from the impact point (2) in the post drop tower tests are very similar to those in previous PMHS tests in a, b, and c.

**Figure 7:**
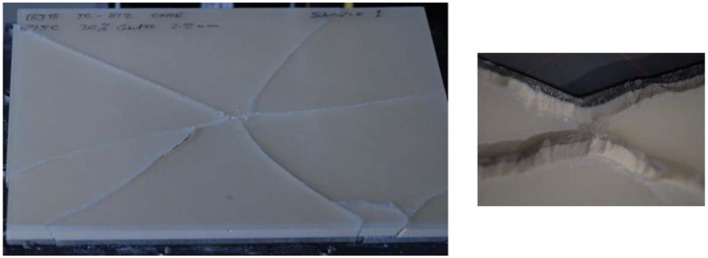
**A typical post drop tower test sample of EPON 815C with 30% milled glass fiber tables and a BJB-TC 812 foam surrogate diplöe**.

When the flat-plate samples were examined for failure, it was discovered that the type of foam material used for the diplöe governed, to a large degree, the type of fracture seen in the tests. Figure 6 shows the type of fracture seen in material C with the U.S. Foam 16# modified foam core with an as formed density of 1.03 g/cc. This was typical of failures seen in either of the materials made with the U.S. Foam 16# core, i.e., B or C. The types of linear ductile cracks seen match those seen in pendulum impacts of skulls from Gurdjian et al. (1950) and Delye et al. (2007). However, the types of fractures produced in using BIB-TC 812 foam resulted in a brittle type of fracture as shown in Figure 7. Therefore, the material combinations that were thought to produce the best cranial surrogate material, not only in terms of force to fracture, but also in terms of the type of fracture produced would be materials B and C, the material consisting of EPON 815C + 30% milled glass fiber surrogate table with the U.S. Foam 16# or modified 16# surrogate diplöe core.

In order to verify that this material is the best to use for the skull surrogate, drop tests are planned using a hemispherical test sample. This will assist in verifying that the force to fracture of a hemispherical sample is much greater than that of a flat-plate and puts the fracture loads more in the vicinity of those seen for intact skull impact testing in the literature. Furthermore, it will support establishing that the fracture characteristics seen in the surrogate skull model are the same as those seen in intact human skulls under impact.

## Conclusion

A cranial bone surrogate has been developed with the bending strength of human cranial bone and having cranial tables with the fracture toughness and tensile strength of the human cranial bone tables. These materials consist of an epoxy resin with a filler (EPON 815C + 30–35% milled glass fiber) for the surrogate cranial tables and foam materials (U.S. Foam 16# and BJB-TC 812) for the surrogate diplöe core. The bending strength equivalent to 8 mm thick, three-layer human cranial bone could be achieved using these materials if the cranial tables were 2 mm thick and the diplöe were 4 mm thick. Flat plates were formed by using these materials and drop tower tests performed. Although the force required to fracture the plates was lower than the force to fracture an intact human skull (due to the absence of skull curvature), the fracture patterns observed were similar to those seen in the literature when intact human skulls were subjected to pendulum impact tests. The materials that best replicated the fracture types seem human skulls under blunt impact from the flat-plate drop tower tests consisted of EPON 815C + 30% milled glass fiber for the surrogate cranial tables and U.S. Foam 16# for the surrogate diplöe core. The next step in the process is to fabricate full hemispherical models using these materials, instrument the models with strain gages, and perform drop tower and/or ballistic impact tests.

## Conflict of Interest Statement

The authors declare that the research was conducted in the absence of any commercial or financial relationships that could be construed as a potential conflict of interest.

## Footnotes

^1^Standard test method for tensile properties of plastics. s.l.: ASTM International, 2008. D 790-07.

^2^Standard test methods for plane-strain fracture toughness and strain energy release rate of plastic materials. s.l.: ASTM International, 1999 (Reapproved 2007). D 5045-99.

^3^Standard test methods for flexural properties of unreinforced and reinforced plastics and electrical insulating materials. s.l.: ASTM, International, 2007. Standard D 790-07.
